# The Effect of Subcutaneous Dexamethasone to Reduce Edema and Ecchymosis in Rhinoplasty Patients

**DOI:** 10.1155/2022/3054767

**Published:** 2022-02-14

**Authors:** Mahboubeh Jafari, Mojtaba Maleki Delarestaghi, Hesam Jahandideh, Shahin Rajaeih, Sara Ghashghaei, David A. Wood

**Affiliations:** ^1^ENT and Head & Neck Research Center, The Five Senses Institute, Iran University of Medical Science, Tehran, Iran; ^2^Department of Otolaryngology Head and Neck Surgery, Firoozgar Hospital, Iran University of Medical Science, Tehran, Iran; ^3^Medical School, Shiraz University of Medical Sciences, Shiraz, Iran; ^4^DWA Energy Limited, Lincoln, UK

## Abstract

**Background:**

Rhinoplasty is one of the most common types of cosmetic surgery undertaken. In most rhinoplasty patients, an osteotomy is used to reshape the nasal pyramid. The most common complications following osteotomy are edema and ecchymosis. Edema and ecchymosis have a significant effect on a patients' satisfaction with surgery and their return to social activities. For this purpose, various methods have been used to reduce edema and ecchymosis, including intravenous injection of corticosteroids, cold compresses, and tranexamic acid.

**Objective:**

To reduce edema and ecchymosis in rhinoplasty patients by administering a subcutaneous injection of dexamethasone and thereby prevent unwanted systemic side effects of corticosteroid treatments.

**Method:**

We conduct a hospital-based nonrandomised study of rhinoplasty patients, with their informed consent treated over the course of one year. Dexamethasone was injected on one side of consenting patient's face immediately before surgery and the results were compared with the opposite side that was not injected. The face images of patients were taken on the front view on the first, third, seventh, and fourteenth days following the treatment. The grade of edema and ecchymosis encountered in each patient was determined by three ENT specialists. The degree of edema and ecchymosis was compared on the injected and noninjected sides and the findings were statistically analysed. The nonrandomised study considered 42 rhinoplasty patients. The mean age of patients was 27.9 years and their age ranged between 17 and 52 years. For 20 patients (47.6%), injection was performed on the right side, and for 22 patients (52.3%), injection was performed on the left side. *Findings*. The statistical analysis of patient outcomes reveals that a supraperiosteal injection of dexamethasone was not effective in reducing edema and ecchymosis after rhinoplasty.

## 1. Introduction

### 1.1. Problematic Outcomes Associated with Rhinoplasty

The nose is the most important organ in facial beauty; for this reason, various surgeries have been performed throughout history to modify the shape in attempts to improve the appearance of the nose [1]. The critical role of the nose in face aesthetics is due to its central location [2].

A common type of nasal cosmetic surgery is rhinoplasty, which requires interventions to positionally manipulate the bone, cartilage, and soft tissue [3]. Due to the forces required as part of rhinoplasty to reshape the nose, in 10% of cases, skin and soft tissue complications occur [4]. Rhinoplasty is divided into two categories, including open surgery or external technique and close surgery or endonasal technique [5]. Ecchymosis (the discoloration of the skin as a consequence of subsurface bleeding associated with bruising) and edema (swelling resulting from fluid trapped in tissue surrounding the nose) tend to be the common complications following open or closed rhinoplasty. Consequently, efforts made to prevent ecchymosis and edema are priorities for the medical team and the rhinoplasty patients [6]. In particular, osteotomy conducted to reshape the bony pyramid of the nose tends to cause damage to the angular arteries and frequently leads to edema and periarticular ecchymosis [7]. Such traumas occur because surgery is conducted in the densely vascularized nasal region [8–10].

Different types of osteotomy are conducted as part of distinct rhinoplasty treatments. All types of osteotomy can result in associated edema and ecchymosis [11]. Lateral osteotomy is a common rhinoplasty procedure. It is typically performed to regenerate the nasal form (pyramid shape). It can be performed in both open and closed configurations to shape the nasal bone. There are two methods employed to conduct lateral osteotomy: (a) linear endonasal (from inside the nose) and (b) percutaneous (external or perfusion-form methods) [11].

The rate and magnitude of edema and ecchymosis vary from patient to patient, even when applying the same techniques and/or taking the same length of time to perform the surgery [8]. In addition to adverse visual consequences, edema and/or ecchymosis tend to prolong recovery times after surgery and delay a patient's return to work and normal social activities.

Moreover, such outcomes lead to anxiety and patient dissatisfaction with the results. Avoidance of edema and ecchymosis after surgery, or fast recovery from it, is clearly beneficial for the patients [9]. Edema sometimes precipitates vision problems early in the postoperative period.

Severe ecchymosis associated with hyperpigmentation following on from inflammation sometimes causes a black ring to form around the eye [10, 12]. Both lateral and periosteal arteries are damaged during lateral osteotomy, thereby leading to edema and ecchymosis [13].

Surgical accuracy acts to limit tissue impacts and reduce negative effect on the blood vessels. However, it is almost impossible to fully prevent edema and/or ecchymosis [14]. The severity of these effects early in the postoperative period are considered likely to affect the aesthetic outcomes of the procedures [15]. Edema and ecchymosis also tend to be uncomfortable for the patient in addition to slowing down the recovery rate after surgery [16, 17], thereby increasing patient anxiety and/or dissatisfaction with the surgery. Edema can delay the healing process of the affected tissues, and ecchymosis may lead to permanent skin pigmentation [18].

Various methods have been employed to reduce edema and ecchymosis following rhinoplasty. These methods include the application of corticosteroids [19], injections of lidocaine and epinephrine solution (typically in a ratio of 1 : 100,000) [16], cold compresses [18], and intravenous injections of tranexamic acid [20]. The objective of all of these treatments is to reduce bleeding during surgery by creating vasoconstriction and inhibiting fluid extravasation, thereby reducing edema and preorbital ecchymosis [21]. None of these treatments provide perfect solutions as they tend to be associated with systemic effects. Treatments that involve topical application of drugs are often favoured, which are easier to use, especially topical, and effectively reduce edema and ecchymosis. They can be used normally in clinical situations.

### 1.2. Expression of the Problem

Studies have examined various methods to reduce edema and ecchymosis in patients undergoing rhinoplasty. Corticosteroids are one of the most commonly used drugs to reduce edema and ecchymosis [4, 6]. Due to their anti-inflammatory effects, corticosteroids prevent the onset of the inflammatory process, including lymphocyte migration, fibrin accumulation, vasodilation, and phagocytic activity [22]. However, various studies recommend the use of dexamethasone as a more favourable alternative to antisteroid drugs due to their higher comparative potency, half-life, and cost-effectiveness. According to Tuncel et al. [21], dexamethasone has the highest anti-inflammatory properties, with a rapid onset of action and a half-life of 54–36 hours. Moreover, steroid use has its own risks such as psychosis, irritability, hypertension, weight gain, uncontrolled blood glucose, and avascular necrosis of the hip [23].

In previous studies, to achieve these goals, different corticosteroids applied in a range of doses have been used as intravenous injections before or after surgery and their effects have been evaluated [6]. Due to the multiple side effects of systemic use of corticosteroids, this study was performed to evaluate the effect of topical dexamethasone in reducing edema and ecchymosis in patients undergoing rhinoplasty. If dexamethasone is found to have a positive effect on reducing edema and ecchymosis associated with rhinoplasty procedures, it offers the attractive possibility of providing a suitable method with less complications than corticosteroids. This study was designed and performed because dexamethasone has not previously been used topically in any of the studies that evaluate and compare its effects with those of corticosteroids in reducing edema and ecchymosis in patients undergoing rhinoplasty.

### 1.3. Novelty of This Research

Due to the effective role of edema and ecchymosis in patient dissatisfaction after rhinoplasty, we have designed and conducted a hospital-based study sought to evaluate alternative ways to reduce edema and ecchymosis in patients following rhinoplasty surgery. The evaluation of the effects of supraperiosteal dexamethasone administered with the aim to reduce edema and periorbital ecchymosis in patients following rhinoplasty has not previously been reported.

### 1.4. Detailed Objectives of Study

Reducing edema and ecchymosis as soon as possible following surgery plays a key role in the patient satisfaction and their ability to return to their normal social activities. Therefore, this study has three objectives:

1. Evaluating the extent of preorbital edema on the injected and noninjected sides of trial patients' faces;
2. Evaluating the extent of preorbital ecchymosis on the injected and noninjected sides of trial patients' faces;
3. Determining whether supraperiosteal dexamethasone injections are effective in reducing edema and ecchymosis in rhinoplasty patients. If so, this offers a viable alternative to the systemic use of corticosteroids for that purpose, thereby avoiding the side effects associated with corticosteroid applications.

## 2. Previous Research Associated with Relevant Dexamethasone Usage

A double-blind study conducted in Turkey in 2006 by Gürlek et al. evaluated the effect of various corticosteroids and tenoxicam on edema and ecchymosis in rhinoplasty patients. 40 patients were divided into 5 groups. One group received 8 mg of betamethasone immediately before surgery, another group received 8 mg of dexamethasone, another group received 40 mg of methylprednisolone, another group received 20 mg of tenoxicam, and the final group received a placebo. Drugs for each group were repeated up to three doses daily. Photographic images were taken of each patient on day 1, day 3, and day 7 following surgery to assess edema and ecchymosis development. The results showed that there was no difference in the degree of edema and ecchymosis between different groups. This led to the conclusion that steroids used in such doses had no effect on reducing edema and ecchymosis [24].

A follow-up study in 2009 involving 40 patients assessed another five groups of rhinoplasty patients. One group received a single 250 mg dose of methylprednisolone immediately before surgery. Another group received a single dose of 500 mg of methylprednisolone. Another group received four doses each of 250 mg of methylprednisolone. Another group received 5 doses each of 500 mg of methylprednisolone. The final group received a placebo. A significant reduction in edema and ecchymosis was observed in the single high-dose methylprednisolone group [25].

Koc et al. conducted a study involving 40 patients in Turkey in 2011, which they split into two groups. One group was administered a single dose of methylprednisolone 1 mg/kg intravenously before surgery. The other group received 3 mg/kg dose intravenously. No significant difference in edema and ecchymosis developments were observed between the two groups. However, a significant reduction in edema and ecchymosis development was observed in both groups compared to a control group. Beneficial outcomes were therefore apparent in both of the groups that received corticosteroids in that study [26].

Also conducted in Turkey, Tuncel et al. in 2013 examined the effect of administering dexamethasone combined with controlled hypotension applied in an attempt to reduce intraoperative bleeding and postoperative edema and ecchymosis. That study involved the evaluation of 60 patients. One group of patients was given a 10 mg/kg dose of dexamethasone intravenously before surgery. Another group was given two such doses, one dose at the beginning of the operation and the other dose 24 hours after the operation. Another group was administered three doses: the first injected at the beginning of the operation; the second before osteotomy; and the third 24 hours after the operation. Edema and ecchymosis developments in the three groups were all observed to be much less than the control group at seven and ten days after surgery. The third group had much less developed edema and ecchymosis on the fifth and seventh days after surgery than the other groups [21].

A 2014 study by Gutierrez and Wuesthoff designed to evaluate the effect of dexamethasone on reducing edema and ecchymosis after rhinoplasty compared to a group administered a placebo. The results of that study showed no significant differences in reducing edema and ecchymosis between two groups receiving different doses of dexamethasone and the control group [18]. In a 2015 study by Valente conducted in Brazil, 42 patients were studied in two separate groups. One group of 20 patients received intravenous dexamethasone injection. The other group of 22 patients served as a control group. Patients were photographed one week after surgery and the results were evaluated by five plastic surgeons. The results identified that periorbital edema and ecchymosis developments on the seventh day after surgery were much lower in the group that had received intravenous dexamethasone than in the control group [27].

A study conducted by Mehdizadeh et al. in 2017 investigated the effects of dexamethasone and tranexamic acid separately and in combination on edema and ecchymosis in rhinoplasty patients. In that study, 60 patients were divided into 4 groups. The first group was injected with dexamethasone, the second group with tranexamic acid, the third group with both, and the control group received a placebo. The study revealed positive effects of reducing edema and ecchymosis in rhinoplasty patients who received dexamethasone and tranexamic acid separately and simultaneously compared to the control group [28].

A study in 2018 by Sanober et al. was conducted on 60 rhinoplasty patients in Pakistan. In that study, one group of patients received 8 mg of intravenous dexamethasone before surgery and the second group received the same dose of dexamethasone 4 hours after surgery. The results of that study identified that the group that had received dexamethasone before surgery displayed less developed edema and ecchymosis than the other group [29]. In 2020, Bian et al. conducted a systematic review and meta-analysis focused on preoperative intravenous dexamethasone. They determined that administering preoperative intravenous dexamethasone did have an effective role in reducing edema and ecchymosis after rhinoplasty [30].

None of the previous studies have examined the effect of administering topical dexamethasone in an attempt to reduce edema and ecchymosis after rhinoplasty surgery, thereby justifying the basis and objectives of the current study.

## 3. Method

### 3.1. Study Group

Forty-two patients at Firoozgar Hospital (Iran University of Medical Sciences, Tehran, Iran) who received rhinoplasty surgery by Dr. Maleki during 2019 and 2020 form the population group considered for this nonrandomised study. Patients with underlying diseases such as hypertension, diabetes, hemolytic diseases, and other specific diseases, as well as patients treated with anticoagulants and antiplatelets or other specific drugs, were all excluded from the study.

### 3.2. Treatment Administered

To evaluate the effect of dexamethasone on edema and ecchymosis after rhinoplasty, an injection of 1 cc of dexamethasone plus 4 cc of lidocaine and epinephrine with a concentration of 1 : 100,000 was administered in preorbital supraperiosteal area and randomly on one side of the patient's face.

### 3.3. Data Collection

The results were compared with the opposite side of the face of the same patient that was injected with 5 cc lidocaine and epinephrine at a concentration of 1 : 100,000 but crucially without dexamethasone. Lateral osteotomy was performed internally for all patients.

Photographs of patients' faces were taken on the first, third, seventh, and fourteenth days after surgery. The degree of edema and ecchymosis on both sides of the face was determined separately by three ear, nose, and throat (ENT) specialists who were not aware of the injection site.

### 3.4. Data Analysis

The McNemar test [31] is used to compare edema and preorbital ecchymosis on both sides of patients' faces. An average grade was established from the comments of the three ENT specialists concerning the degree of edema and ecchymosis for each patient. Those average results were then compiled and statistically assessed using SPSS software [32].  Table 1 presents classifications of each grade of edema and ecchymosis. Based on that, for edema, grad 0 presents no edema, grade 1 represents mild edema, grade 2 follows moderate edema, grade 3 illustrates severe edema, and grade 4 shows eyelid swollen shut. Furthermore, considering ecchymosis, grade 0 offers no ecchymosis with no colour change, grade 1 has the significance of yellowish colour difference, grade 2 possesses the symptom of light purple, and grade 3 is associated with dark purple, as well as grade 4 with very dark purple. Based on this analysis, all gradings were achieved available in Tables 2 and 3.

### 3.5. Ethical Considerations

Before entering the study, the patients were informed about the details of the study plan and the reason for conducting the study. The possibility of asymmetry of edema and ecchymosis occurring on alternate sides of the face was explained to the patients. On that basis, they were asked to provide (or not) their written consent to participate in the nonrandomised study.

## 4. Results

In this study, 42 patients were studied and the mean age of the patients was 27.88 years with a standard deviation of 9.98 years. The oldest patient was 52 years old and the youngest was 17 years old.

Three patients (7.1%) were male and 39 patients (92.9%) were female. The mean age of male patients was 28.67 with a standard deviation of 3.78 and the mean age of female patients was 27.82 with a standard deviation of 10.33 years. This difference was not statistically significant (*p*=0.89). For 20 patients (47.6%), the injection was performed on the right side of the face, and for 22 patients (52.3%), the injection was performed on the left side of the face.

**Figure 1:**
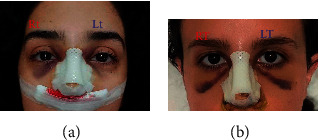
Example of grading assessment for two patients A and B.

Figure 1 provides an instance of two patients. As can be seen, patient A possesses grade 3 and 2 for edema and ecchymosis, respectively, in the left side; however, patient A has grade 3 and 3 for edema and ecchymosis, respectively, in the right side. Furthermore, patient B possesses grade 1 and 4 for edema and ecchymosis, respectively, in both left and right side as the same.

**Figure 2:**
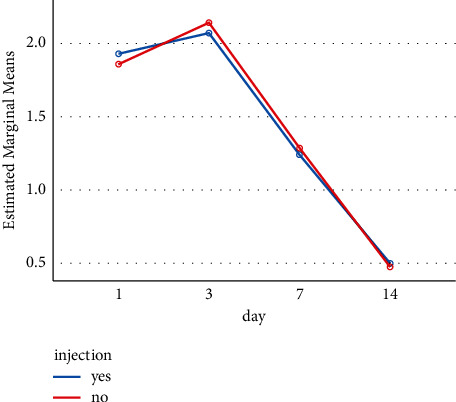
The reduction of edema on days 1, 3, 7, and 14 based on having or not having an injection. According to the graph, the reduction of right edema after 14 days was statistically significant in both groups (*p* < 0.001). But no significant difference was observed between the groups (*p*=0.95).

**Figure 3:**
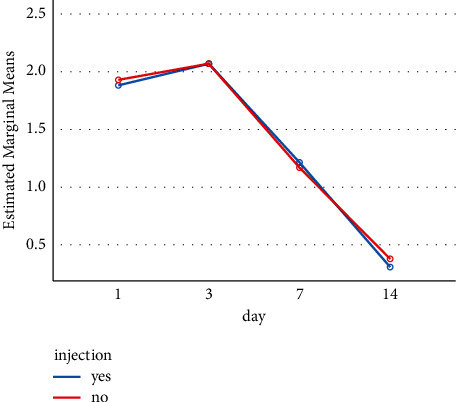
The reduction of ecchymosis on days 1, 3, 7, and 14 based on having or not having an injection. According to the reduction chart, the rate of reduction after 14 days in both groups was statistically significant (*p* < 0.001). But no significant difference was observed between the groups (*P*=0.92).

Table 2 shows the frequency distribution of edema grade on days 1, 3, 7, and 14 after administering the injection. Two categories are distinguished: one covering the parts of the face receiving the injection and one covering the parts of the face not receiving the injection (Figures 2 and 3).

The results and their analysis (Figures 2 and 3) indicate that supraperiosteal dexamethasone injection did not have a positive or negative effect on edema and ecchymosis after rhinoplasty surgery for the 42 patients studied. In both injected and uninjected cases (opposite sides of the same face), the rate of edema and ecchymosis on day 14 was significantly reduced but the dexamethasone injection did not appear to play any role in that development.

## 5. Conclusion

According to the statistical analysis and evidence reviewed from the results of this nonrandomised study, subcutaneous dexamethasone has no positive or negative effect on edema and ecchymosis development in rhinoplasty patients in the first two weeks after surgery. Differences in the rate of improvement in edema and ecchymosis 14 days after surgery on the side of the face where dexamethasone was injected and the opposite side of the same face receiving no injection were found to be statistically insignificant. These results indicate that the use of subcutaneous dexamethasone injections is not worthwhile as part of rhinoplasty procedures.

## Figures and Tables

**Table 1:** Description of grades used for the assessment.

Diagnosis	Grading	Description
Edema	0	No edema
	1	Mild edema (minimal)
	2	Moderate edema (extending to the iris)
	3	Severe edema (covering the iris)
	4	Eyelid swollen shut
Extent of ecchymosis	0	No ecchymosis
	1	Up to medial one-third of the eyelid
	2	Medial half of the eyelid
	3	Entire eyelid
	4	Extending to the lateral canthus
	5	Extension of ecchymosis below the malar bone

**Table 2:** Frequency distribution of edema grade on days 1, 3, 7, and 14 after injection, classified into those cases of the patients' faces that received the injection and those cases of the patients' faces that did not receive the injection.

Time after surgery, day	Grade	Number of cases with injection	Number of cases without injection	*p*
The first day	0	1	1	0.96
	1	9	11	
	2	24	23	
	3	8	7	
The third day	1	8	11	0.28
	2	23	15	
	3	11	15	
	4	0	1	
The seventh day	0	4	2	0.71
	1	25	28	
	2	12	10	
	3	1	2	
The fourteenth day	0	22	23	0.97
	1	19	18	
	2	1	1	

**Table 3:** Frequency distribution of ecchymosis grade on days 1, 3, 7, and 14 after injection classified into those cases of the patients' faces that received the injection and those cases of the patients' faces that did not receive the injection.

Time after surgery, day	Grade	Number of cases with injection	Number of cases without injection	*p*
The first day	0	2	2	0.88
	1	14	12	
	2	17	18	
	3	5	7	
	4	4	2	
The third day	0	4	6	0.85
	1	11	11	
	2	11	7	
	3	10	10	
	4	6	7	
The seventh day	0	14	19	0.68
	1	14	9	
	2	6	4	
	3	7	8	
	4	1	1	
The fourteenth day	0	32	30	0.92
	1	8	9	
	2	1	2	
	3	1	1	

## Data Availability

The data used to support the findings of this study are included within the article.
